# Consumer Perception and Acceptability of Lupin-Derived Products: A Systematic Review

**DOI:** 10.3390/foods12061241

**Published:** 2023-03-14

**Authors:** Bruno Abreu, João Lima, Ada Rocha

**Affiliations:** 1 Faculty of Nutrition and Food Sciences, University of Porto, Rua do Campo Alegre, 823, 4150-180 Porto, Portugal; adarocha@fcna.up.pt; 2 Scientific-Pedagogical Unit of Dietetics and Nutrition, Coimbra Health School, Polytechnic Institute of Coimbra, 3046-854 Coimbra, Portugal; joao.lima@estesc.ipc.pt; 3 ciTechCare—Center for Innovative Care and Health Technology, Rua de Santo André, 2410, 2410-541 Leiria, Portugal; 4 GreenUPorto—Sustainable Agrifood Production Research Centre, Campus de Vairão Edifício de Ciências Agrárias (FCV2), Rua da Agrária, 747, 4485-646 Vairão, Portugal

**Keywords:** lupin products, food innovation, consumer studies, sensory analysis

## Abstract

The addition of lupin into other foods can enhance their nutritional value and may be an acceptable approach to introducing lupin into the food supply, particularly as an ingredient. Lupin could be used in many food products (bakery products, pasta, beverages, meat products and dairy products) to improve their protein content and possible nutraceutical effects. The main aim of this study is to summarise the recent formulation trends with lupin as an ingredient of new food products based on consumer perception and acceptability. The present systematic literature review was conducted through the Preferred Reporting Items for Systematic Reviews and Meta-Analyses guidelines. The eligibility criteria for the articles to be considered were: (a) the manufacturing of a food product with lupin as a formulation ingredient; (b) the food product developed was tested by a sensorial panel. A total of 33 studies filled the inclusion criteria and were incorporated into the qualitative synthesis. The sensory analysis of each product was notoriously different based on the jury evaluators and measurement scales used but revealed high acceptability rates for possible future consumers. The high protein and fibre contents of lupin were the most cited reason associated with the importance of nutrient-rich food products for consumers. More research on foods with high nutrition profiles and well-established sustainability parameters is crucial to promote healthier food environments.

## 1. Introduction

The bioavailability of nutrients and bioactive compounds present in food is assumed to be an important factor by policymakers, consumers, and industry which strongly value functional food production for environmentally friendly food products [1,2]. With this need for global food on the horizon, the role of legumes has a significant preponderance [3]. Dietary guidelines’ main objectives are to simplify the interpretation of healthy dietary patterns for a specific population. Legumes are difficult to position due to their nutrient composition, being high in dietary protein, fibre and micronutrients—part of the reasons why research supports the beneficial effects of legumes on cardiovascular health, diabetes, and obesity [4,5,6]. The new Danish nutritional guidelines highlight legume dishes cooked from scratch, frozen, dried or canned in their own guise to reduce meat consumption [7]. Although the recent American dietary guidelines finally recognised the benefits of “pulses”, they still label the unclear grouping of legumes with different food groups and other foods that have completely different nutritional profiles (such as oilseeds) in the same category, contributing to the current knowledge gap by being a source of confusion for consumers [8]. An example of the correct scientific information translation to the general population is the Mediterranean diet, which appeals to the legume intake and, more precisely, the Portuguese food guide (*Roda dos Alimentos*), which focuses on a portion dedicated to daily legume consumption [9].

Legumes are part of the *Fabaceae* or *Leguminosae* family, which designate the low-fat content legumes from edible seeds which are harvested for dried grains [10,11]. Legume nutrient-dense profile, with a focus on the high-fibre content, is assumed to be the key reason for the indirect positive outcomes on cardiovascular risk factors and gut microbiota activity [6]. Currently, the primary markets for legume seeds are of limited use in human foods linked to unhealthy ultra-processed products, wasting a potential source of nutritious and health-promoting food ingredients [10]. Although the recognition of legumes is increasing, their potential for utilisation as ingredients for food product development is not being completely exploited due to sensory aspects because of their inherent flavour, taste and aroma sensations which are often perceived as negative features by consumers [11,12,13,14], as well as the occurrence of intestinal discomfort when ingested becoming a frequent excuse for their exclusion from the daily intake [5,6,15].

In addition, legumes may trigger severe allergic reactions; peanuts and soybeans are two of the eight foods marked as significant food allergies and must be carefully handled when added to a product. Other types of legumes, such as lentils, chickpeas, green beans, peas or lupin, deserve similar special attention as a precaution [16,17]. It also compromises various factors which can lower their nutritional value, the so-called “antinutritional factors”, such as lectins, enzyme inhibitors, tannins, oxalates, or phytates [18]. Soaking is the treatment most commonly applied to destroy unwanted compounds in the soaking water to inactivate enzymes or to destroy antinutritional factors [11,18,19].

An undervalued legume that addresses all the issues mentioned before is the lupin bean (*Lupinus* spp.), native to the Mediterranean region, North Africa and Latin America [3,10,11,20,21,22,23,24]. Four major cultivated species gained more relevance to be utilised in human food production for their low levels of alkaloids contained in their seeds: *Lupinus albus* (typical white lupine), *Lupinus luteus* (the yellow lupine), *Lupinus angustifolius* (known as blue lupin or narrow-leafed lupin) and *Lupinus mutabilis* (common pearl or Andean lupin) [18,20,22,23,24,25,26,27,28,29,30].

Regarding the pedo-climatic conditions, lupin has great adaptability and can be grown in very different ecological conditions [22]. Lupin crops are well adapted to grow in low temperatures and acidic and meagre soils, so they are most determinant in zones where other agricultural crops struggle [24,31]. As legumes, they can also play an important role in replenishing soil nitrogen in concrete by fixing atmospheric nitrogen into nitrate, a usable form of nitrogen by the following crop [24,32]. Therefore, lupin cultivars are considered crucial to crop rotation figures.

Lupin grain composition depends on the specific specie, but in general, it is assumed to be high in protein content varying between 30–42% and dietary fibre from 30–41%, mainly insoluble, while it is low in starch content and gluten-free [20,21,22,25,33,34,35]. The fat content mainly consists of mono and poly-unsaturated fats, and omega 3, 6 and 9 fatty acids [33,35]. They are rich in minerals like iron, magnesium, zinc, calcium and potassium and also contain vitamins and antioxidants, such as carotenoids, B complex vitamins and tocopherols [21,22,33,35].

In addition, when compared to most other legumes, lupin stands out for having low levels of antinutritional aspects, such as phytic acid, alkaloids, oligosaccharides and lectins [35,36]. Newly developed sweet lupin varieties have substantially decreased and are seen only in trace amounts [36], specifically, for not having in its constitution trypsin inhibitors and saponins [21,27]. The decrease of these factors to very low levels is achieved through different techniques such as germination, cooking, maceration, fermentation or selective extraction [35,37].

In scientific literature, potential relationships are categorised according to the form of lupin consumed. When referring to whole lupin consumption, there is evidence that it improves satiety, lowers blood pressure and improves glycaemic control. On the other hand, consuming lupin protein and isolated fibre demonstrated the strongest positive results for reducing blood pressure and serum lipids [25,26,33,34,38,39]. The supporting evidence suggests that lupin is equally (or possibly more) effective than other legumes in protecting health in the long term [33,34,39].

The safety concerns of lupin ingredients include the development of biogenic amines and the presence of allergens. Lupin bean allergy is still relatively unusual; only a limited number of adverse events associated with the ingestion have been reported, but cross-reactivity between peanut and lupin occurs. Therefore, conglutins, such as -α, -β, -γ and -δ, are candidate lupin allergens. Considering the high severity of allergic reactions to peanuts, the cross-reactivity of new lupin derivatives must be carefully assessed, and commercially processed lupin products must be properly labelled to minimise the danger for potential allergic consumers [26,30,35,40].

The addition of lupin into other foods can enhance their nutritional value and may be an acceptable approach to introducing lupin into the food supply, particularly as an ingredient [33]. This indicates that lupin could be used in many food products (bakery products, pasta, beverages, meat products and dairy products) to improve their protein content and possible nutraceutical effects [21,25]. Foods based on sweet lupin proteins are gaining attention from industry and consumers due to their health and environmental role [3,27]. There is an enormous potential market demand for lupin-based products, with niches in growing sectors, such as vegetarians, vegans, and people with intolerance or allergy to gluten, soya, milk or eggs.

However, despite lupin’s nutritional and health-promoting benefits, its applications in food manufacturing are still rather limited [25,29,41,42]. The lack of thickening and gelling functionality has limited its use as a human food ingredient [30]. Specifically, few studies research the utilisation of methods to improve lupin protein functionality. New modification methods must improve techno functionality (solubility, viscosity, gelling, foaming, emulsification) as well as bio functionality (nutritional, digestibility and hypoallergenic properties) by altering the structure of proteins in all conformation levels [30,43]. It is also necessary to improve the sustainability of most of these processes, namely in terms of water consumption [34]. Hopefully, the relation with new and affordable food sources as a trend, where lupin is considered a potential protein source, is clearly in expansion [19,44].

The main aim of this study is to summarise the recent formulation trends with lupin as an ingredient of new food products based on consumer perception and acceptability. This systematic review has been conducted to critically evaluate and compare novel studies on lupin-derived products, giving special consideration to the chemical composition and nutritional, sensory and physiochemical evaluation.

## 2. Materials and Methods

The present systematic literature review for selection and results analysis was conducted through the Preferred Reporting Items for Systematic Reviews and Meta-Analyses (PRISMA) guidelines. The checklist of 27 items recommended for reporting in systematic reviews was meticulously completed in order to produce the outcomes for this work [45]. The protocol for this work was published in advance and is publicly available in PROSPERO with the register number CRD42022379036 [46].

### 2.1. Eligibility Criteria

The question for this research was, “Are lupin-derived products well accepted from the perspective of consumers?” Lupins have been used as food for over 4000 years. The first papers on the development of lupins to be used in food products and their sensory analyses date back to the 1960s [22,23,24]. For this particular reason and the recent significant development of this line of research, this study intends to focus on research trends over the last 5 years. The papers were excluded in this review if they did not meet the following criteria: (a) the publication date is after 1 January 2018; (b) the document type is article excluding, therefore, proceeding papers, meeting abstracts, etc.; (c) the source type for publication are scientific journals; (d) the publication language of full text is English. Concerning the eligibility criteria for the articles to be considered on the topic, the following criteria were outlined: (a) the manufacturing of a food product with lupin as a formulation ingredient; (b) the food product developed was tested by a sensorial panel.

### 2.2. Search Strategy

The papers were searched on a 5-year timeline (from 1 January 2018 until 21 October 2022) in the following databases: Web of Science, Scopus and PubMed. The search used keywords concerning lupins with food innovations, and that included consumer perception, such as sensory evaluation. The following keywords with the respective Boolean operators were applied: “lupin*” (to include “lupin”, “lupine”, “lupinus” and “lupin bean”) AND “product*” (to include “product innovation”, “product development” and “product trends”) AND (“consumer*” (to include “consumer acceptance”, “consumer liking” and “consumer perception”) OR “sensory*” (to include “sensory quality”, “sensory characteristics”, “sensory properties” and “sensory evaluation”).

### 2.3. Selection and Data Collection Processes

Research results were imported via the EndNote 20^®^ reference management and bibliography software (EndNote 20, Clarivate Analytics, Philadelphia, PA, USA), and duplicates were removed. The papers were screened in two logical steps, first, by reading the title and its abstract, followed by reading the full text. In order to select the papers by the authors in a blind way, the list of papers was inserted in Rayyan^®^ intelligent systematic review software (Rayyan, Rayyan Systems Incorporated, Boston, MA, USA), and each one of them was selected individually. From then on, there was a meeting between the three main authors to discuss the conflicted papers marked for inclusion by at least one of them. A data extraction form was inserted in Microsoft Excel^®^ (Microsoft Office Home & Business, Version 16.66.1, Redmond, Washington DC, USA) spreadsheet to include firstly the studies origin, reference, lupin specie and which constituent of lupin was used, and secondly, food product innovation, sensory panel description, sensory analysis outcomes, innovative processing or functionality used for food production and reasons that lead to the insertion of the product in the market.

## 3. Results

### 3.1. Eligibility Criteria

**Figure 1:**
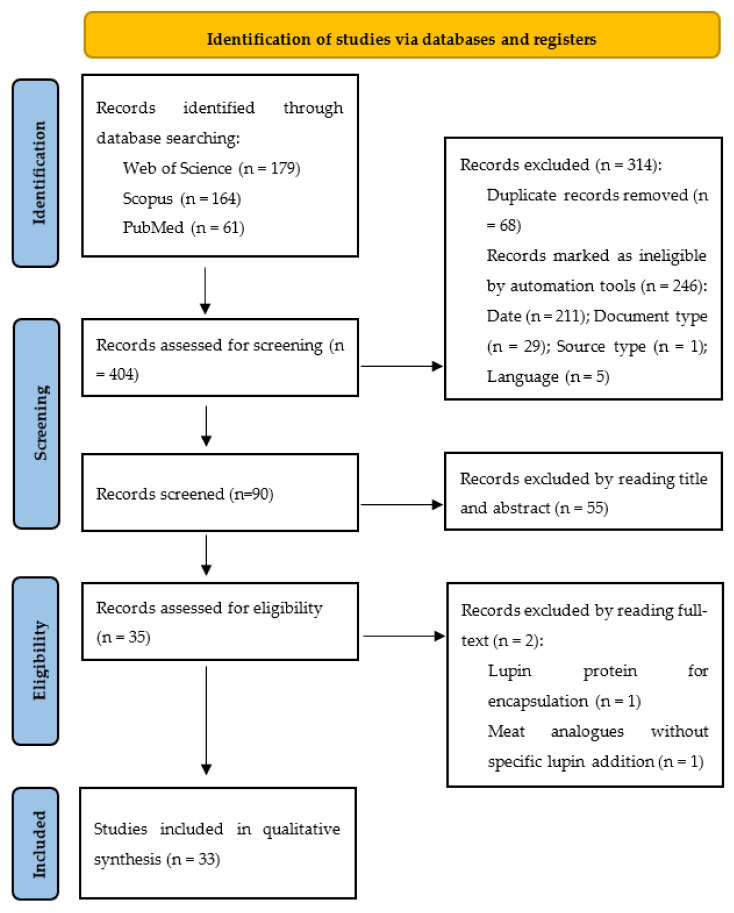
Flow diagram of Preferred Reporting Items for Systematic Reviews and Meta-Analyses (PRISMA) for study selection.

The search was carried out on 21 October 2022 and identified a total of 404 records. By reading the articles’ titles and abstracts, 371 papers were immediately excluded. The main reasons for exclusion were previously filtered by automation tools that included the date (after 2018), document nature (scientific articles), source type (academic journals) and language (English mandatory) of the manuscripts. Through the automatic removal of duplicates by EndNote 20, 35 papers remained. Thus, the process led to the full text read of the 35 articles, and from those, two were excluded. The explanations for the exclusion of these papers were using lupin protein solely for encapsulation purposes (n = 1) and legume-based meat analogues produced without specific lupin addition (n = 1). A total of 33 studies filled the inclusion criteria and were incorporated in the qualitative synthesis for this systematic review (Figure 1).

### 3.2. Study Characteristics

The characteristics and major outcomes of the selected studies are presented in Table 1. Thereby, the research data collected is summarised by the description of the food products developed with the nomenclature used by the authors, their formulation ingredients and nutritional composition according to information provided in each paper, the sensory analysis, information about the subjects tested, the scales used and the outcomes for each of them and the processing used for the lupin incorporation in the product. Whenever it was impossible to represent some of the desired information, the cell was marked as “not specified”. Each item was tried best to be the closest possible to the terminology expressed by the authors in the original papers.

### 3.3. Results of Syntheses

In order to synthesise the results obtained in a brief and accessible way, the principal characteristics of the selected studies are illustrated in Table 2. It is designated by the country of lupin acquisition, the specific *Lupinus* spp. used, the product type developed by category and the part of lupin used for processing.

## 4. Discussion

The systematic review regarding the recent lupin research for human consumption and their sensory analysis observed a range of results across combinations of ingredients that directly influenced the nutritional profile of the food products. In the 33 articles that fit the selection criteria, the processes for lupin incorporation usually included soaking in water to improve the nutritional properties, and then seeds were transformed according to their usefulness. The sensory analysis of each product was notoriously different based on the jury evaluators and measurement scales used but revealed high acceptability rates for possible future consumers. After categorising the studies by the aforementioned and general classification, such as geographical and biological information, product types created, and form of lupin utilised, this review noted relationships between them, expressing these specific details in the following paragraphs.

Starting by describing the lupin production figures in the last 5 years according to the Food and Agriculture Organization of the United Nations statistics available publicly [80], Oceania gets the pole position by having the majority (57.8%, 713 k ha.), followed by Europe which comfortably occupies second place (32.1%, 396 k ha), then Africa (5.4%, 66 k ha), with America having close data (4.6%, 57 k ha); in last place is Asia (<1%, 108 ha). From the geographical point of view, it is possible to group the included articles by origin continents: Europe [50,51,53,55,59,63,70,72,73,75], South America [54,58,61,66], Asia [47,56,60,64,65,68,69,74,77,78], Africa [49,52,62,67,71,76,79] and Oceania [48,57]. This grouping revealed that Europe and Asia are, for now, the leading promoters of these novel food products, and Africa also reports many studies. Comparing the figures numbers within the grouping of this study, it is seen that the agricultural production is not proportional to the scientific production of this theme. This data could indirectly show that developed and developing countries are committed to modifying the food systems for a healthier and more sustainable future. However, prudence must be taken because it is interpreted as an excessive assumption since much more scientific literature is needed to assume that.

Another relation based on the lupin species used when discriminated is possible by comparing its country of acquisition: *Albus* [47,49,52,53,59,62,64,65,67,68,69,71,72,73,74,75,76,77], *Angustifolius* [48,51,55,57,67,75], *Mutabilis* [54,61,66] and *Luteus* [58]. *Lupinus albus* is commonly used in Europe, Asia and Africa, with the highest appearance rates in the selected studies, followed by *Lupinus angustifolius* in Oceania. The utilisation of *Lupinus mutabilis* and *Lupinus luteus* was exclusively associated with South America. This information provides the possibility of using various species of lupin to develop food products according to the country of origin, pointing to the interest in comparing similar products from different species [67,75].

To divide the products developed by categories, the following terms were determined: meat alternatives [47], dairy alternatives [48,49,50,51,52,53,54,55], grains [56,57,58,60,61,62,63,64,65,66,67,68,69], snacks [70,71,72,73,74,75,76] and desserts [77,78,79]. The grain category has the most results, and it can be subdivided into terms such as bread making [56,57,58,59,60,61,62], enriched pasta [63,64,65,66] and traditional foods [67,68,69]. In accordance with this type of subdivision, dairy alternatives can also be labelled as cheese [48], yoghurt [49,50,51] and milk [52,53,54,55] analogues. It can be argued that lupin-based meat analogues are a gap in the industrial market and within the scientific community that is expected to be improved in the near future.

On the pretext of consumption of lupin as a legume that contains excellent amounts of protein, fibre and bioactive compounds and therefore contributes to improving health, several studies have analysed micronutrients and minerals contents, polyphenolic content and antioxidant activity of developed food products [52,56,58,60,62,65,67,68,69,70,72,77,78]. However, very few discussed the health benefits of its consumption, and those who did failed to explain them in detail [52,58,60,69,78]. Future research should consider the consumption of this type of food product to agree with the health benefits proven by studies of the consumption of whole lupin and lupin as supplements.

Different processes were used to transform the lupin for food product formula incorporation, such as flour [52,56,57,58,59,61,64,65,66,67,68,69,70,71,72,73,74,76], ground [48,78,79], powder [47,49,60,62,77], liquid fraction [51,53,54] and isolate protein [50,55,63,75]. Although there were similarities between flour and ground processing explanation, different terminologies were used according to the nomenclature used by the original authors. In most studies, an initial phase of soaking seeds in water to improve nutritional properties (to break down phytic acids and anti-nutrients to improve digestion and diminish alkaloid content) was common. Most works report the de-hulling of lupin seeds before starting their processing. To promote the circular economy in food systems and the manufacturing of innovative foods, some studies utilised the hull of lupin that is usually discarded as residual leftover [56,57,58,64,65,69,74,76].

The only patent clearly expressed in the included studies was a lupin protein isolate produced by aqueous extraction and isoelectric precipitation from *Prolupin GmbH* [50,63,75]. None of the other papers expressed concerns about trademark search in their results. This is an essential process for enhancing viable products in the marketplace, which must be reflected in future research addressing this topic. Even though research on lupin addition in food products for human consumption goes back to the 1960s [22,23,24], there is not a significant retail volume nowadays in the market, mostly because of the lack of funding and consumer awareness. The contributions of authors such as Yaver et al. [56,64,65,69] and Aslan et al. [77,78], with several published articles, must be recognised once the importance of this theme emerges.

The sensory appeal evaluation of foods is essential to classify their acceptance. It is well established that a small panel of judges is sufficient to perform a reliable trial hedonic test. In the studies selected, the majority used less than 75 individuals to carry out the tests [48,50,51,52,53,54,55,56,57,59,60,62,63,64,65,67,68,69,70,71,72,73,74,75,76,77,78,79]. However, it is considered that larger groups of up to 150 individuals [61,66] and larger than 200 [58] give a more accurate prediction of consumer acceptance in the global market. In this direction of translating the scientific data to the practical field, it is also important to describe the sample of judges according to age, sex or health status, but only in less than half of the articles selected was this description provided [51,54,55,56,58,65,66,67,69,73,75,76,77,78].

Different methodologies with diverse scales were used in the studies to perform the sensory analysis. Sensory tests provided reliable information about the relationship between exposure and judges’ acceptance of lupin products, which indirectly supports the evidence for consumer studies. The most-used scale was the hedonic tests, nonetheless with different dimensions: 5-point [48,52,53,58,68,70,74], 7-point [56,67,77,78,79] and 9-point [47,49,54,59,60,61,62,64,65,66,69,72,76]. Uncommon scales of 0 to 10 [51,55,57,63] and 0 to 100 [71,75] were used, as well as descriptive methods [52,64]. It should be known that a mixture of sensory methods provides a more in-depth inciteful knowledge between the perception of sensory attributes and their influence on liking or disliking. Combining hedonic, discrimination, and descriptive tests can reveal fundamental associations between the sensory profile and consumer liking [49,53,60,62,77].

Several studies used sensory assessments to evaluate numerous samples of the same product with different formulations. Relative to these comparisons of different formulation samples, the average range identified was 15% of lupin addition. Therefore, the authors suggest 15% of lupin addition as a cut-off value in novel food products whenever sample numbers are limited. The selected articles presented results based on different ratios: some suggest less than 10% of lupin addition [49,56,59,62,65,67], others 15% of lupin addition, considered a middle value [52,60,64,71], and more than 20% of lupin addition [54,61,68,70,74,76,78]. Future studies may benefit from conjoining sensory methods with instrumental analysis to better understand the physical attributes that determine the liking and disliking of lupin-derived products.

The reasons for inserting each novel food product in the market were expressed in all articles selected. The high protein and fibre contents of lupin were the most cited reasons associated with the importance of nutrient-rich food products for consumers [49,51,52,53,54,55,56,58,59,60,61,63,65,67,68,69,70,71,74,75,76,77,79]. The argument for the economical and good cost-beneficial food product development rate was also mentioned [47,52,62,79]. Besides the most general arguments, it also reflects the importance of these food products for specific populations like celiac with the need for varied, acceptable and reasonable gluten-free products in the market [58,60,62,64,66,69,70,72] and milk-allergic people for the dairy alternatives developed [48,51,79], as well as alternative non-omnivorous food patterns who abstain from eating animal-based foods, such as vegans and vegetarians [48,50,78,79].

## 5. Conclusions

To the best of the authors’ knowledge, this is the first systematic review to investigate the manufacturing of lupin-derived products and their sensory analyses combined. This review found high acceptability rates for future consumers of novel food products with lupin in their formula ingredients. Lupin and its use in food manufacturing reveal a possible wide utilisation in different food product types, such as meat alternatives, dairy alternatives, snacks and desserts. The present work highlights that more research should be done in order to improve the manufacturing of plant-based products for awareness of the current food choice pattern and to improve sustainability in food systems. More research on foods with high nutrition profiles and well-established sustainability parameters is required in scientific literature, and this data is crucial to promote healthier food environments. In the particular case of this study, lupin was the main ingredient considered as an interesting approach at both levels to improve the food products available in the market. Since the attributes of perception of products determine their acceptance, sensory analyses should be done whenever possible to create those.

## Figures and Tables

**Table 1:** Characteristics and major outcomes of studies exploring lupin use for food product formulations.

Reference	Food Product Developed	Formulation Ingredients	Nutritional Composition (g/100 g Dry Weight Basis or %s)	Subjects (n) for Sensory Analysis	Sensory Analysis Scale Used and Outcomes	Processing/Functionality Used for Lupin Incorporation
**MEAT ALTERNATIVES**						
[47]	Camel burgers with lupin powder	Camel meat, lupin, garlic, a mixture of spices (black pepper, coriander, cumin, desserts and nutmeg) and salt	21.6 g protein 6.2 g fat 10.2 g carbohydrates 0.8 g fibre	Not specified	9-point hedonic scale Juicer (7.2), coolness (7.2), flavour (7.7), colour (7.6), textures (7.7) and general acceptance (7.9) Good rate of all sensory characteristics studied	Lupin seed powder Seeds were processed and ground; the sample was kept frozen until used
**DAIRY ALTERNATIVES**						
[48]	Lupin cheese	Lupin milk and different coagulations (vinegar, lemon juice, starter culture or rennet enzyme)	27.3 g protein 9.9 g fat 4.0 g carbohydrates	20 trained panellists (10 males and 10 females)	5-point hedonic scale Appearance (4.4), colour (4.2), flavour (4.2), texture (4.2), overall acceptability (4.7) Lupin cheese produced from PBA Jurien using vinegar was the most acceptable	Lupin seed ground Seeds were cleaned, broken into halves, and the hull removed with mortar and pestle; Seeds were soaked overnight in water and then ground with a stainless-steel gas-tight blender
[49]	Fat-free stirred yoghurt with lupin hull powder	Fat-free cow milk and lupin husk powder	Not specified	Not specified	9-point hedonic scale Appearance (8.4), consistency (8.8), flavour (8.2) and overall acceptance (8.8) The addition of 1% lupine hull scored the highest sensory characteristics	Lupin hull powder The hull was removed from the seeds and passed through a lab hammer mill; Grounded hull fibres were then re-milled and passed through a 500 μm sieve; the sample was packed and kept under refrigeration
[50]	Plant-based yoghurt alternative	Skim milk powder, whey protein isolate, lupin protein isolate, coconut oil, anhydrous milk, fat, sucrose and distilled water	Not specified	30 untrained panellists	Sorting task With a protein ratio of 67:33, were characterised as pleasant and non-homogenous; The others with 50:50 were described as unpleasant and bitter The ones with anhydrous milk fat were described as milky and “goaty”; The others containing coconut oil were characterised as fruity and fresh	Lupin protein isolate Isolate was purchased already processed
[51]	Yoghurt and milk analogues prepared from lupin	Lupin liquid fraction, lactic acid bacteria starters and barley starch	Not specified	14 trained panellists (7 males and 7 females) and 22 untrained panellists (9 males and 13 females, 21–61 years old)	Line scale of 0 to 10 intensities Appearance yellowness (4.3), appearance homogeneity (6.5), odour intensity (3.8), odour vinegar (2.8), odour beaniness (2.9), flavour intensity (6.3), flavour beaniness (3.3), sweetness (1.1), sourness (5.4), bitterness (3.1), umami (2.6), astringency (3.9), texture graininess (2.3) and texture thickness (3.3) 9-point hedonic scale Colour (6.3), appearance (6.2), odour (5.0), flavour (4.2) and overall (4.5) The sample with *Lactococcus lactis* and *ssp. Lactis* was the most accepted by the two panels	Lupin liquid fraction Seeds were first rinsed and soaked overnight in excess tap water; after de-hulling and rinsing, soaked seeds were ground with tap water using a cutter to form a slurry; The slurry was filtered through a washable and reusable fine mesh plastic cheese filter inserted in a screen bowl of centrifuge at 4000 rpm; Centrifugation took 10 min, allowing easy and hygienic collection of both liquid and solid fractions
[52]	Oat-based with lupin and stinging nettle fermented beverage	Oat, lupin, stinging nettle and premix flour (black cardamom, malted wheat, pumpkin, spiced chilli peppers and table salt)	19.9% protein 10.1% fat 66.9% carbohydrates 3.6 g fibre	50 untrained panellists.	5-point hedonic scale Taste (4.2), appearance (4.2), aroma (3.5), mouth feel (3.6), consistency (4.5) and overall acceptability (4.3) The blending of 15% lupin resulted in the best overall sensory acceptance	Lupin seed flour The cleaned lupin was first roasted for 10 min in a metal pan; after cooling, the roasted lupin grain was soaked for 8 days changing the water twice a day; lupin was de-hulled and sun-dried before grind in a lab mill and passed through a 0.5 mm sieve
[53]	Legume beverage from chickpea and lupin	Lupin, chickpeas, green and yellow peas	5.4 g carbohydrates	29 untrained panellists	5-point hedonic scale Colour (3.8), appearance (3.9), taste (4.0), flavour (3.1), consistency (3.3) and overall appreciation (3.0) The legume mixture-based beverages with both chickpea and lupin evidenced the highest sensory characteristics	Lupin liquid fraction The dried seeds were soaked twice in warm tap water (30–35 °C) and once in cold tap water (15–20 °C) for 16 h; the water was removed, and the soaked seeds were cooked for 30 min after boiling in a pressure pan with fresh tap water; the sample was drained, and fresh tap water was added; the mixture was milled in the food processor at 20,500 rpm for 4 min
[54]	Functional beverage with milk, tarwi and oatmeal	Fresh milk, de-bittered lupin grains, oatmeal, honey bee, bottled water and probiotic culture	3.6 g protein 0.2 g fibre	30 untrained panellists (20–30 years old)	9-point hedonic scale Overall acceptability (8.5) The major perception of sensory characteristics was found in the sample with 30% lupin	Lupin liquid fraction Grains were combined with water and homogenised in an industrial blender at 3600 rpm for 5 min; then, the sample was filtered using a stainless-steel mesh and sterile gauze cloth, separating the retained solids from the supernatant to obtain a homogeneous solution; the solution was pasteurised at 85 °C for 15 min, cooled, and stored in refrigeration at 4 °C
[55]	Fermented protein isolate hydrolysates	Lupin isolate protein, enzymes (cysteine endopeptidase, serine endopeptidase and aspartic endopeptidase) and water	74.8% protein	10 trained panellists (healthy)	Scale from 0 to 10 Oatmeal-like (3.8), cocoa-like (6.2), malty (5.9), green grassy (2.3), pea-like (5.8), fatty (2.1), cardboard-like cucumber-like (2.8), roasty (3.0), cooked potato-like (1.6) and earthy (2.6) The sample with *Lactobacillus sakei carnosus* was found on the positive side and was the best rated	Lupin isolate protein Seeds were de-hulled, separated and passed through a roller mill; the resulting flakes were de-oiled in *n*-hexane and extracted with hydrochloric acid for 1 h; Suspension was separated using a decanter centrifuge at 4 °C for 1 h, and the supernatant was discarded; the acid pre-extracted flakes were dispersed in sodium hydroxide for 1 h at room temperature while stirring and separated by centrifugation at 4 °C for 1 h; the precipitated proteins were separated by centrifugation for 130 min and neutralised with sodium hydroxide, pasteurised at 70 ° C for 10 min and spray-dried with an inlet temperature of 180 °C and an outlet temperature of 80 °C
**GRAINS**						
[56]	Cereal-legume flour blend	Wheat, rye, barley, oat, chickpea, soybean, lupin, yeast, water and salt	12.9 g protein 1.3 g fat	25 untrained panellists (22–48 years old)	7-point hedonic scale Symmetry (7.0), pore structure (6.9), taste (7.0), odour (6.7), appearance (6.7) and overall acceptability (7.0) The highest overall acceptability scores were obtained using 5% of flour developed in breads	Whole lupin seed flour Lupin was milled (<500 μm) using a hammer mill into whole grain flour with a 100% extraction rate
[57]	Germinated and fermented lupin flour	Lupin, soybean and flour	Not specified	5 trained panellists	10-point computerised time-intensity scale (0–350) for odour intensity Beany green (175), floral (0), meaty (250), nutty (50), woody green (225), sweet (175), baked (75) and mushroom soil (225) Germination significantly affected the aroma profile of lupin	Lupin seed flour After removal of damaged material, seeds were sanitised in hydrogen peroxide solution and rinsed with water until a neutral pH was obtained; seeds were soaked in water for 8 h; then, seeds were placed on trays covered with germination paper and germinated for 72 h in 22 °C and humidity 50–60% with access to natural daylight (12 h) and darkness (1 h); soaked and germinated samples were dried in a cabinet dryer for 18 h at 50 °C, and the temperature was increased by 10 °C every hour until 80 °C; after cooling to room temperature, dried seeds were ground using a lab mill (0.5 mm sieve); finally, stored in sealed air-tight packs at 4 °C until analysis
[58]	Bread dough	Lupin grit flour, lupin hulls flour, wheat flour and flaxseed expeller four	11.1% protein 2.5% fat 43.6% carbohydrates 6.3% fibre	259 untrained panellists (5 categories based on age: 20–29 years old, 30–39 years old, 40–49 years old, 50–59 years old and >60 years old; and based on gender: 147 female and 112 male)	5-point hedonic scale Like extremely/like/neither like nor dislike/dislike/dislike extremely Colour (60%/31%/5%/2%/2%) flavour (56%/36%/5%/1%/2%), texture (64%/25%/10%/1%/0%) and overall acceptability (60%/35%/4%/1%/0%) The individuals under 40 years old showed a slightly lower acceptance than the ones over that age	Whole and hulled lupin seed flour The lupin seeds were de-hulled, and the kernel and hulls were milled separately (<0.3 mm particle size) with a lab mill; the obtained was to be incorporated afterwards as flours on the different flour blends
[59]	Bread dough	White wheat flour, lupin flour, compressed yeast, salt and water	Not specified	30 semi-trained panellists	9-point hedonic scale Appearance (8.0), colour (7.9), taste (7.9), smell (8.5), texture (7.5), flavour (6.5) and global acceptability (8.5) A 10% of lupin flour addition in wheat flour had the highest effect of improving the sensory characteristics	Lupin seed flour The germination process was performed at a constant humidity of 80% and 25 °C in dark conditions; seed grains with rootlets were freeze-dried to lower the moisture using a lyophiliser at −50 °C and 10 Pa for 24 h; Then, seeds were ground with a lab mill
[60]	Multigrain pan bread	Quinoa, lupin, fenugreek, yellow maise and psyllium	17%g protein 6.4% fat 59.1% carbohydrates 15.2% fibre	10 trained panellists	9-point hedonic scale Taste (6.5), odour (6.5), texture (7.0), crust colour (6.5), crumb colour (6.5), appearance (7.5) and overall acceptability (6.9) The fortification with 15% of lupin positively influenced the acceptance	Lupin seed powder Lupin seeds were soaked in water for 12 h to remove bitterness; Seeds were germinated for 3 days in an incubator at 25 °C; the seeds were dried to obtain a fine powder, and the sample was stored at 5 °C
[61]	Wheat bread	Wheat flour, de-bittered lupin sweet flour, yeast extract, guar gum, salt, sugar, fat, bread-improver and water	12.9 g protein	112 untrained panellists	9-point hedonic scale Appearance (7.1), flavour (7.0), texture (7.1) and overall liking (7.0) A 20% substitution of lupin flour in wheat flour caused a good sensory evaluation	Lupin seed flour Flour was purchased already processed
[62]	Gluten-free flatbread	Rice flour, lupin powder, sweet potato powder, millet flour, salt, baking powder, sunflower oil and corn oil	10.4% protein 3.3% fat 83.6% carbohydrates	10 untrained panellists	9-point hedonic scale Appearance (8.2), crust colour (8.5), crumb colour (8.3), texture (8.3), taste (8.6), odour (8.7) and overall acceptability (8.6) Using sweet lupin powder to 10% proved the highest acceptability	Lupin seed powder Firstly, seeds were carefully cleaned and freed from broken seeds and extraneous matter; then, they were soaked in water for 12 h, soaked water was discarded; seeds were cooked in boiling water for 10 min and manually de-hulled and dried in a drying oven at 45–58 °C overnight (18–20 h); The result obtained was milled using a lab mill followed by sieving to obtain a fine powder and then packed in polyethene bags and kept for further analyses
[63]	High-protein hybrid pasta	Wheat semolina, buckwheat flour, faba bean flour, lupin protein isolate, sodium chloride and water	Not specified	8 trained panellists	Quantitative descriptive analysis with continuous scales of 10 Flour odour (4.0), legume odour (4.8), beige colour (5.2), flour flavour (5.0), legume flavour (3.8), sweat taste (1.6), bitter taste (1.5), aftertaste (2.8), elasticity (4.3), hardness (2.9), chewiness (3.0), adhesiveness (2.5), overall quality (8.3) The results obtained suggest high consumer acceptance	Lupin isolate protein Isolate was purchased already processed
[64]	Enriched pasta	Durum wheat, semolina, de-bittered lupin flour, phosphorylated cross-linked wheat starch, vital wheat gluten and microbial transglutaminase	Not specified	12 untrained panellists	9-point hedonic scale Colour (8.3), taste (8.1), odour (7.5), appearance (8.3), stickiness (8.6) and overall acceptability (8.3) The addition of 15% lupin flour proved high sensory analyses results	Whole lupin seed flour Ultrasound application: boiled seeds were soaked in 25 °C water for 60 h and were sonicated for 25 min every 4 h; seeds were dried in a hot-air oven at 50 °C; Then, were ground into whole flour (<500 μm); After that, the flour sample was stabilised by dry roasting method at 160 °C for 30 min
[65]	Enriched pasta	Durum wheat, semolina, de-bittered lupin flour, phosphorylated cross-linked wheat starch, wheat flour, baker’s yeast, salt and water	14.2 g protein 1.8 g fat 13.1 g fibre	12 untrained panellists (25–45 years old)	9-point hedonic scale Colour (7.5), taste (8.2), odour (8.0), appearance (7.4), stickiness (8.2) and overall acceptability (7.8) The sample enriched with 10% lupin flour had the highest overall acceptability score	Whole lupin seed flour Ultrasound application: bitter seeds were boiled in water for 75 °C; seeds were soaked in 25 °C water for 60 h and were sonicated for 25 min every 4 h with an ultrasonic probe in a glass beaker; the soaking water was changed every 4 h during the soaking; de-bittered seeds were dried in a hot-air oven at 50 °C; then, they were ground (<500 μm) using a hammer mill into whole lupin flour with a 100% extraction rate; after that, the flour sample was stabilised by dry roasting method at 160 °C for 30 min in the hot-air oven
[66]	Gluten-free pasta with lupin flour	Rice flour, whole eggs, lupin flour and guar gum	18.6% protein 7.0% fat 62.7% carbohydrates 0.7% fibre	112 consumers (62 males and 50 females)	9-point hedonic scale Flavour (6.2), texture (5.9), overall appearance (6.1), overall results (6.1) The most accepted sample was the one with 20 g of lupin flour	Lupin seed flour De-bittered flour was purchased already processed
[67]	Injera from tef complemented with lupin	Tef grain, lupin, water and ersho	15.5% protein 2.8% fat 76.9% carbohydrates	50 untrained panellists (20 males and 30 females)	7-point hedonic scale Colour (6.2), texture (5.7), taste (6.0), rollability (5.8), no eye (5.7), eye size (5.8), eye distribution (6.0), top and bottom surface (6.0), aroma (6.2) and overall acceptability (6.2) The sample developed with the addition of 10% lupin flour had the highest overall acceptability score	Lupin seed flour The de-bittering process for the seeds consisted of cleaning, boiling and de-bittering; seeds were boiled in water for 50 min to destroy thermolabile antinutritional factors and to soften the seeds’ hulls; the boiled lupine seeds were de-bittered with water at room temperature; The soaking water was changed every 12 h for 144 h; Afterwards, the whole seed was de-hulled manually, and the kernel was dried at 105 °C for 3 h in an oven; the seeds were dried and milled into a fine powder by using a disk attrition mill; then, they were sieved with a sieve size of 750μm and packed in polyethene bags, kept for further analyses at 4 °C
[68]	Tarhana soup	Wheat flour, buckwheat, quinoa, lupin, full-fat concentrated set yoghurt, bakers’ yeast, peeled and chopped dry onions, tomato paste, red pepper and salt	19.7 g protein	25 trained panellists	5-point hedonic scale Taste (4.5), odour (4.7), colour (4.6), grittiness (4.6), sourness (4.0) and overall acceptability (4.6) More than 20% lupin flour incorporation produced negative effects on the acceptability	Lupin seed flour Seeds used after grinding to <500 μm size
[69]	Gluten-free tarhana soup	Corn flour, rice flour, legumes (chickpea, common bean, lentil, soybean and lupin) flour, yoghurt, tomato paste, onion, baker’s yeast, ground paprika and salt	Not specified	6 untrained panellists (30–55 years old)	9-point hedonic scale Taste, odour, colour, consistency, mouthfeel and overall acceptability The sample with lupin flour was not subjected to sensory evaluation because of lower technological characteristics than other legume samples	Whole lupin seed flour Dried de-bittered seeds were ground to <500 μm size by a lab hammer mill
**SNACKS**						
[70]	Biscuits	Lupin flour, wheat flour, vegetable shortening and powdered sugar	Not specified	12 trained panellists	5-point hedonic scale Round shape stability after baking (5.0), colour intensity (2.2), odour intensity (2.5), odour intensity of legumes (1.0), flavour intensity (2.0), flavour intensity of legumes (2.2) and acceptability (4.5) 25% of lupin flour addition was considered the most appropriate	Lupin seed flour Not specified
[71]	Semi-hard biscuits	Soft wheat flour, whey protein, lupin, margarine, baking powder, sugar, milk, egg and salt	13.2% protein 13.0% fat 71.2% carbohydrates	10 trained panellists	100-degree test with 5 factors (0–20) Colour (19.3), crust appearance (18.7), texture (18.3), aroma (19.0), taste (18.3) and overall acceptability (93.7) The sample with 15% lupin powder had the highest score of acceptance	Lupin seed flour Lupin seeds were powdered using a custom electric mill
[72]	Gluten-free biscuits and salty crackers	Rice flour, lupin, corn starch, sugar, butter, salt, tartaric acid and water	Not specified	15 trained panellists	9-point hedonic scale A high sensory score (7.9) obtained in the biscuits sample with 40 g of lupin flour A high sensory score (7.7) was obtained in the crackers sample with 40 g of lupin flour	Lupin seed flour Seeds were ground fine in a domestic mill, and the flour was filtered with a 0.4 mm filter
[73]	Crackers of legume flour	Wheat flour, chickpea flour and lupin flour, water, canola oil, baking powder, salt and sugar	Not specified	24 untrained panellists (10 males and 14 females) 22 untrained panellists (5 males and 17 females)	Flash profile First session: each judge creates a self-list of attributes Other sessions: all attributes were pooled into a single list and presented to the judges Sensory attributes generated and used by more than one judge: crispy (6), floury (4), sweety (4), roasted chickpea (3), dietary (2), fatty (2), legumes (2) and neutral (2)	Lupin seed flour Flour was purchased already processed
[74]	Chips	Lupin flour, corn flour, whole wheat flour, salt, various spices (thyme, red pepper and sunflower oil), guar gum and monosodium glutamate	Not specified	10 trained panellists	5-point hedonic scale Taste (3.2), crispness (3.3), colour (3.5), odour (3.3), oiliness (3.5) and general appearance characteristics (3.7) The most liked formulation for overall general acceptability was the fried ones with 50–60% of whole lupin flour	Whole and hulled lupin seed flour Lupin seeds were dried at 40 °C for 10 h in two different forms, hulled manually peeled and whole grain; after drying, lupin seeds were milled using a mechanical mill to obtain flour which was passed through a 0.5 mm sieve before further use
[75]	Legumes and Pseudocereal protein snacks	Lupin protein isolate, lentil protein isolate, faba bean concentrate, pseudocereal flours, wheat starch, maise starch and pea starch	Not specified	15 trained panellists (22–54 years old)	Visual analogue scale ranging from 0 to 100 Pea-like (18), fishy (12), cheesy sweaty (15), roasty popcorn-like (7), rancid (37), fatty cardboard-like (25), crunchiness (60), elasticity (35), firmness (95), porosity (46), umami (20), salty (8), preference (18) and overall intensity (55) Lupin protein-based extrudates exhibited satisfactory texture and sensory properties	Lupin isolate protein Isolate was purchased already processed and produced by aqueous extraction and isoelectric precipitation
[76]	Maise-based extruded snack food	Maise flour, lupin flour,	17.8% protein 5.7% fat 64.9% carbohydrates 2.6% fibre	50 semi-trained panellists (19–50 years old)	9-point hedonic scale Colour (7.3), flavour (6.2), texture (6.1), taste (6.2) and overall acceptability (6.8) Overall acceptability scores of the samples demonstrated a significant positive effect of lupin flour addition up to 20%	Lupin seed flour The seeds were cleaned, washed and pre-soaked in water for 12 h; the soaked seeds were boiled for one hour to eliminate heat-sensitive antinutritional factors; The boiled seeds were in a de-bitter process by soaking for 5 days at room temperature; the soaked water was changed every 6 h; then, whole seeds were de-hulled manually, and the seeds were dried at 65 °C for 24 h in hot air oven; the dried samples were milled using a batch miller to a particle size of 0.5 mm, packed in a polyethene bag and stored at 4 °C until use
**DESSERTS**						
[77]	Functional cake	Whole egg, sugar, all-purpose shortening, skimmed milk powder, baking powder, wheat flour, lupin and soy	10.8 g protein 19.0 g fat	12 untrained panellists (25–55 years old, healthy and non-smokers)	7-point hedonic scale Colour (6.9), pore structure (6.6), taste (7.0), odour (7.0) and overall acceptability (6.8) The use of a 10% ratio of lupin increased the overall acceptability scores of the cake	Lupin seed powder Hulls of lupin seeds were removed manually; Seeds were dried in a hot air oven at 65 °C for 8 h; then, they were ground in a hammer mill equipped with a 500 μm sieve
[78]	Sponge cake	Wheat flour, sugar, shortening, cow milk, egg, salt, baking powder, vanilla, lupin, soy, diacetyl tartaric acid ester, monoglycerides, diglycerides and xanthan gum	9.8 g protein 18.8 g fat	12 untrained panellists (25–55 years old)	7-point hedonic scale Colour (7.0), appearance (6.8), pore structure (6.9), taste (6.7), odour (6.9) and overall acceptability (6.9) The substitution of egg with 25% lupin milk had the highest overall score	Lupin seed ground Hulls of seeds were manually removed; Seeds were dried at 50 °C for 12 h before ground; mixed with hot water at 90–95 °C for 15 min; After cooling, it was homogenised again at 10,000 rpm for 1 min; the sample was stored in a refrigerated condition
[79]	Ice cream	Soybean, lupin and cow milk	4.3 g protein 6.3 g fat 21.4 g carbohydrates	15 untrained panellists	7-point hedonic scale Appearance (5.8), taste (5.5), flavour (6.0), texture (6.2) and overall acceptability (6.1) The 50:50 blend ratio of lupin milk to cow milk had the best overall acceptability	Lupin seed ground For 18 h at room temperature, seeds were cleaned, weighed, washed and soaked in water; lupins were repeatedly rinsed and drained with cold water; the amount of water needed to produce the milk was weighed back; the remaining water was used to prepare lupin milk for molten grinding; the mix was then filtered and was boiled for 10 min

**Table 2:** Location of the lupin acquisition, specific lupin species used, product type classification and the form of lupin incorporated into food product formulations in eligible studies.

Country: Lupin Acquisition	Reference	Specific Specie: *Lupinus*	Product Type: Meat Alternative (MA) or Dairy Alternative (DA) or Grain (G) or Snack (S) or Dessert (D)	Lupin Form: Seed (S) or Hull (H) or Isolate Protein (IP)
Egypt	[49]	*Albus*	DA	H
	[71]	*Albus*	S	S
	[62]	*Albus*	G	S
	[67]	*Albus* and *Angustifolius*	G	S
Ethiopia	[52]	*Albus*	DA	S
	[79]	Not specified	D	S
	[76]	*Albus*	S	S
Australia	[48]	*Angustifolius*	DA	S
	[57]	*Angustifolius*	G	S/H
Iraq	[47]	*Albus*	MA	S
Saudi Arabia	[60]	Not specified	G	S
Turkey	[77]	*Albus*	D	S
	[78]	Not specified	D	S
	[68]	*Albus*	G	S
	[69]	*Albus*	G	S/H
	[74]	*Albus*	S	S/H
	[56]	Not specified	G	S/H
	[64]	*Albus*	G	S/H
	[65]	*Albus*	G	S/H
Finland	[51]	*Angustifolius*	DA	S
Romania	[59]	*Albus*	G	S
Germany	[50]	Not specified	DA	IP
	[63]	Not specified	G	IP
	[75]	*Albus* and *Angustifolius*	S	IP
	[55]	*Angustifolius*	DA	IP
Greece	[73]	*Albus*	S	S
Hungary	[72]	*Albus*	S	S
Portugal	[53]	*Albus*	DA	S
Slovakia	[70]	Not specified	S	S
Chile	[58]	*Luteus*	G	S/H
Ecuador	[66]	*Mutabilis*	G	S
	[61]	*Mutabilis*	G	S
Peru	[54]	*Mutabilis*	DA	S

## Data Availability

Bruno Abreu, João Lima, Ada Rocha. Consumer perception and acceptability of lupin-derived products: a systematic review. PROSPERO 2022 CRD42022379036 Available from: https://www.crd.york.ac.uk/prospero/display_record.php?ID=CRD42022379036 (accessed on 10 February 2023).
